# Anticoagulant Activities of Indobufen, an Antiplatelet Drug

**DOI:** 10.3390/molecules23061452

**Published:** 2018-06-15

**Authors:** Jia Liu, Dan Xu, Nian Xia, Kai Hou, Shijie Chen, Yu Wang, Yunman Li

**Affiliations:** 1 Department of Marketing, Hangzhou Zhongmei Huadong Pharmaceutical Company, Hangzhou 310011, China; liujia0918@163.com; 2 State Key Laboratory of Natural Medicines, Department of Physiology, China Pharmaceutical University, Nanjing 210009, China; 1621090709@stu.cpu.edu.cn (D.X.); 18061612181@163.com (N.X.); houkai0624@outlook.com (K.H.); 17351014665@163.com (S.C.); 3 Collaborative Innovation Center for Cardiovascular Disease Translational Medicine, Department of Pharmacology, Nanjing Medical University, 140 Hanzhong Road, Nanjing 210029, China

**Keywords:** indobufen, anticoagulant effect, coagulation factor, platelet factor.

## Abstract

Indobufen is a new generation of anti-platelet aggregation drug, but studies were not sufficient on its anticoagulant effects. In the present study, the anticoagulant activity of indobufen was determined by monitoring the activated partial thromboplastin time (APTT), prothrombin time (PT), and thrombin time (TT) in rabbit plasma. We evaluated the anticoagulant mechanisms on the content of the platelet factor 3,4 (PF3,4), and the coagulation factor 1, 2, 5, 8, 10 (FI, II, V, VIII, X) in rabbits, as well as the in vivo bleeding time and clotting time in mice. The pharmacodynamic differences between indobufen and warfarin sodium, rivaroxaban, and dabigatran were further studied on thrombus formation and the content of FII and FX in rats. Animal experiments showed that intragastric-administrated indobufen can significantly reduce the APTT, PT, TT, PF3, FI, II, V, VIII, and X plasma contents. Its inhibitory effect on plasma FII was better than thrombin inhibitor dabigatran with effect on FX better than FXa inhibitor rivaroxaban. These results suggest that indobufen has some anticoagulant effects as strong as some conventional anticoagulants. The mechanism may be related to both exogenous and endogenous coagulation system.

## 1. Introduction

Blood coagulation is a series of complex chemical chain reactions characterized by initiation, propagation, and termination phases of thrombin generation, and anticoagulants are chemical compounds or proteins that prevent blood from clotting by binding to coagulation factors and preventing them from binding to phospholipid membranes [1]. The coagulation cascade is initiated by two pathways, known as the intrinsic pathway and extrinsic pathway. The intrinsic pathway is initiated by substances within the damaged blood vessel, whereas the extrinsic pathway is activated when blood is exposed to tissue factors from the surface of extravascular cells [2]. Coagulation factors join both the intrinsic and extrinsic pathways of coagulation, in which factors 1,2,5,8,10 (FI, II, V, VIII, X) are important components [3]. The common process of the intrinsic and extrinsic coagulation pathways is to activate the resulting prothrombin (FII) to form thrombin (IIa), which subsequently catalyzes fibrinogen (FI) to fibrin monomers (FIa). FIIa not only converts FI into FIa but also enhances its own generation through the activation of the cofactors FV, FVIII, and FXI in the so-called feedback loop [4]. Studies show that the FXa activation of FV is of paramount importance in initiating the coagulation system [5]. Prothrombin time (PT), activated partial thromboplastin time (APTT), thrombin time (TT), and fibrinogen (FIB) were associated with coagulation, which is frequently used to examine thrombotic diseases [6].

**Figure 1:**
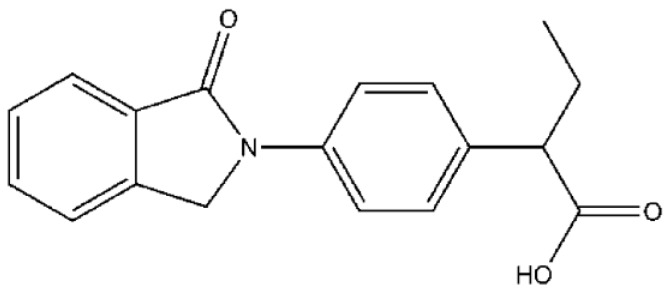
The chemical structure of indobufen.

Indobufen (laboratory code K 3920, Figure 1)—chemically 2-[*p*-(1-oxo-2-isoindolinyl) phenyl] butyric acid—is a new generation of anti-platelet aggregation drug that reversibly inhibits the platelet cyclooxygenase and decreases thromboxane A2 (TXA2) production [7]. It also inhibits platelet aggregation induced by adenosine diphosphate (ADP) [8], epinephrine, platelet activating factor [1], collagen, arachidonic acid [9]. Clinical uses include ischemic stroke [4,10], myocardial infarction [8], non-rheumatic atrial fibrillation [11,12], peripheral vascular disease, and thrombosis formation in patients with venous thrombosis [13], especially in the prevention and treatment of atherothrombosis [14]. Although the mechanism of anti-platelet effects of indobufen has been thoroughly studied in the clinic, little is known about its anticoagulant mechanisms. The existing research results only show that indobufen can improve the blood coagulation function and enhance the erythrocyte deformation ability [15], with no activities on the blood clotting plasma parameters. There are no studies on the effects of indobufen on the coagulation mechanisms, especially coagulation factors.

The available data suggest that indobufen acts as an antiplatelet agent by reducing the levels of platelet factor 3 and 4 (PF3, 4) [7]. Platelet factor 3 is a phospholipid moiety expressed on the cell membranes of activated platelets that takes part in platelet aggregation during clot formation [16]. PF4 is a very abundant platelet α-granule CXC chemokine that is released during platelet activation and works as the binding site of FV [17]. It promotes pro-coagulant activities by preventing the formation of a stable heparin—anti-thrombin III–thrombin ternary complex [18]. Activated platelets not only provide a phospholipid surface for the activation of clotting factors during blood clotting but also release a variety of coagulation factors such as clotting factor 1, 5, 11, 13, and so forth [19]. Therefore, platelets and coagulation factors are closely related to each other and causally interrelated, both participating in the body’s injury repair mechanisms. In this research, we evaluated indobufen from both the impact on the coagulation pathway and the anti-thrombotic function. The experiments focused on the effect of indobufen gavage administration on platelet factors and clotting factors in order to initially examine its coagulation functions in intrinsic and extrinsic coagulation systems. Furthermore, we selected three kinds of commonly used oral anticoagulant drugs for comparison, including the most traditional anticoagulant warfarin sodium (vitamin K inhibitor [1]) and two new oral anticoagulants rivaroxaban (a representative direct FXa inhibitor [20]) and dabigatran etexilate (reversible direct thrombin (FIIa) inhibitor [21]). Although warfarin has a strong anticoagulant effect, its side effect of bleeding is also very strong [22]. Clinically, studies have shown that indobufen is superior to warfarin in reducing the bleeding side effects in patients with atrial fibrillation [12,23]. Meanwhile, FIIa and FXa are among the most important components of both exogenous and endogenous coagulation pathways. The comparison of indobufen with the above commonly used anticoagulants would help us evaluate its specific pharmacological effects and clinical anticoagulation values.

## 2. Results

### 2.1. Indobufen Showed Anticoagulant Activities in Rabbits and Mice

#### 2.1.1. Effects on Activated Partial Thromboplastin Time (APTT), Prothrombin Time (PT), and Thrombin Time (TT)

**Figure 2:**
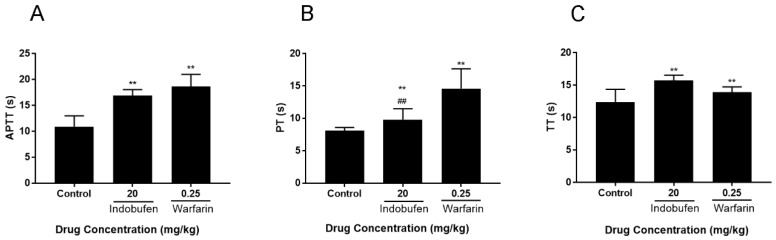
The effects of indobufen on activated partial thromboplastin time (APTT, **A**), prothrombin time PT, **B**), and thrombin time (TT, **C**) in rabbits. ** *p* < 0.01 versus the control group ^##^ *p* < 0.01 versus the warfarin sodium group (mean ± S.D., *n* = 9).

The effect of intragastric-administrated indobufen on APTT, PT, and TT in rabbits are shown in Figure 2. Indobufen significantly prolonged APTT (*p* < 0.01) in rabbits compared with the negative control group, indicating that the anticoagulant mechanism of indobufen is mainly mediated by the endogenous coagulation system. Meanwhile, significantly prolonged rabbit PT (*p* < 0.01) indicating that indobufen can also act on the extrinsic coagulation system, which is significantly better than that of warfarin (*p* < 0.01), and that indobufen has some influence on the coagulation factors. At the same time, significantly prolonged PT in rabbits (*p* < 0.01) revealed that indobufen can slow down the conversion of fibrinogen (FI) to fibrin (FIa) and has an effect on the fibrinolysis system.

#### 2.1.2. Indobufen Increased the Clotting and Bleeding Time in Mice

**Figure 3:**
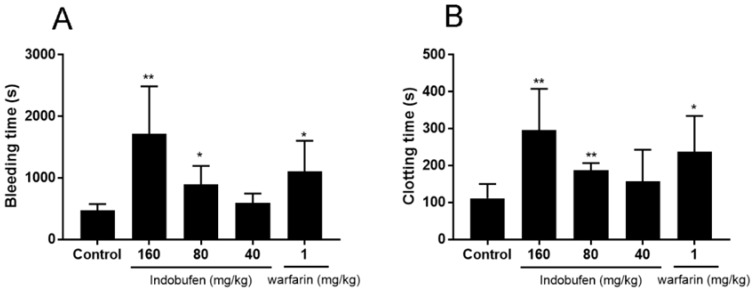
The effects of indobufen on bleeding time (**A**) and clotting time (**B**) in mice. * *p* < 0.05, ** *p* < 0.01 versus the control group; (mean ± S.D., *n* = 10).

The mouse bleeding time assay (Figure 3A) showed that indobufen significantly prolonged the bleeding time in mice at 80 and 160 mg/kg (*p* < 0.01) compared to the negative control group, proving its effects on platelet and capillary related functions. Additionally, compared with the negative control group, indobufen significantly prolonged the clotting time of mice at 80 and 160 mg/kg (*p* < 0.01, Figure 3B), indicating that indobufen has anticoagulant effects, and can act on the endogenous anticoagulant system. 

### 2.2. Effects on Platelet Factor 3 and 4 and Coagulation Factors 1, 2, 5, 8, and 10 in Rabbits

**Figure 4:**
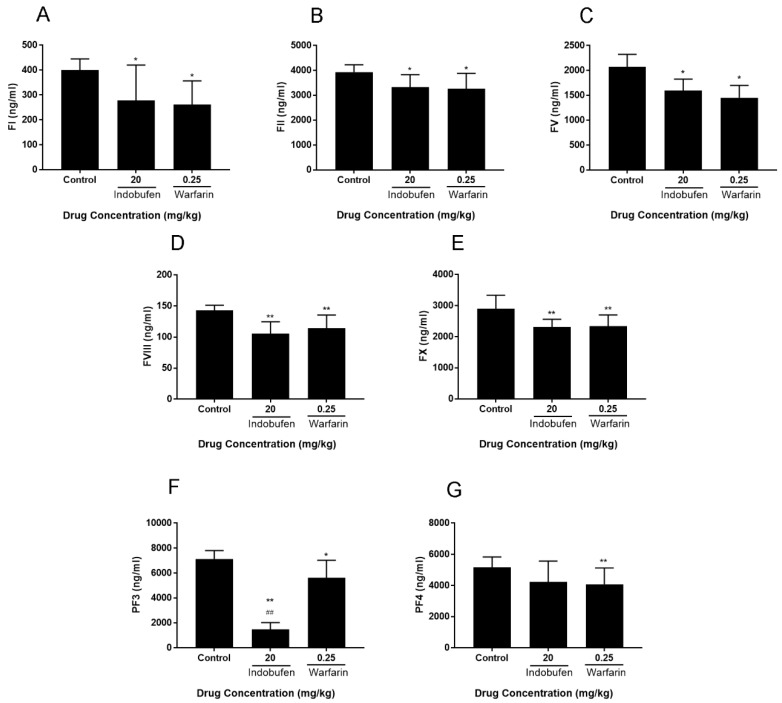
The effects of indobufen on coagulation factor 1 (FI, **A**), 2 (FII, **B**), 5 (FV, **C**),8 (FVIII, **D**), 10 (FX, **E**), and platelet factor 3 (PF3, **F**), 4 (PF4, **G**) in the poor platelet plasma (PPP) of rabbits. * *p* < 0.05, ** *p* < 0.01 versus the control group; ^##^ *p* < 0.01 versus warfarin sodium group (mean ± S.D., *n* = 9).

To explore which components of the coagulation system can be affected by indobufen, we examined its effect on platelet factor 3, 4 (PF3, 4) and coagulation factors 1, 2, 5, 8, and 10 (FI, II, V, VIII, X) in poor platelet plasma (PPP, to eliminate the effect of indobufen on platelet aggregation, Figure 4) in rabbits. The effects of indobufen on clotting factors 1, 2, 5, 8, and 10 were significantly different from that of the negative control group (*p* < 0.05, Figure 4A–E) and were as effective as warfarin sodium. Meanwhile, the indobufen group significantly decreased the levels of platelet factor 3 (*p* < 0.01, Figure 4F), and the effect exceeded warfarin sodium (*p* < 0.01).

### 2.3. Indobufen Inhibited Thrombosis Formation in Rats

**Figure 5:**
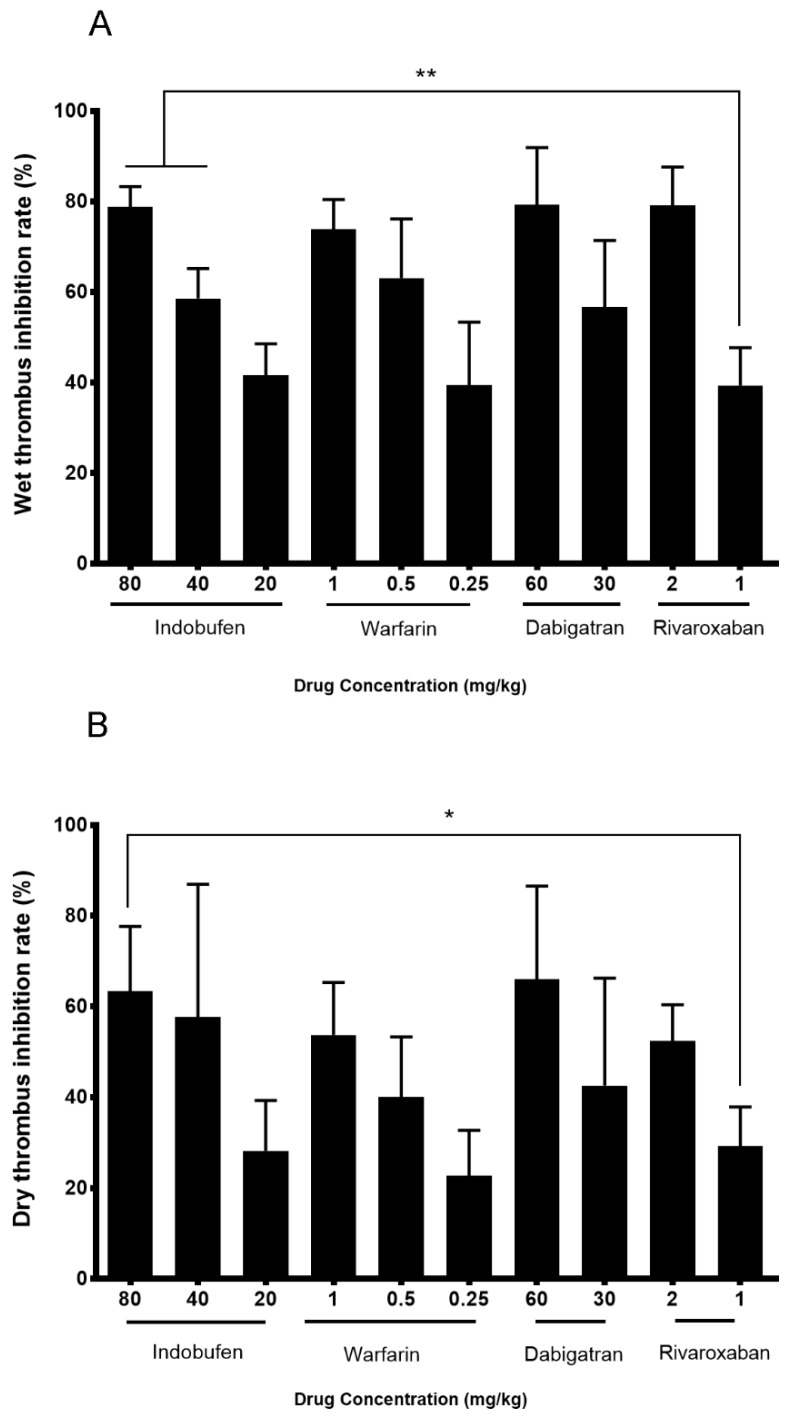
The effects of indobufen on thrombosis formation in a rat model of an arteriovenous shunt. (**A**) The results of wet thrombus inhibition. (**B**) The results of dry thrombus inhibition. * *p* < 0.05, ** *p* < 0.01 versus the rivaroxaban 1 mg/kg group (mean ± S.D., *n* = 8).

Figure 5 shows that compared with the same dose group of warfarin sodium and dabigatran, the indobufen groups had no statistical difference. Compared with 1 mg/kg rivaroxaban, the 40 and 80 mg/kg of the indobufen group significantly improved the rate of the thrombus wet weight inhibition rate (*p* < 0.01) and 80 mg/kg of indobufen significantly improved thrombus dry weight inhibition rate (*p* < 0.05).

### 2.4. Indobufen Reduced the Content of Coagulation Factor 2 in Rats

**Figure 6:**
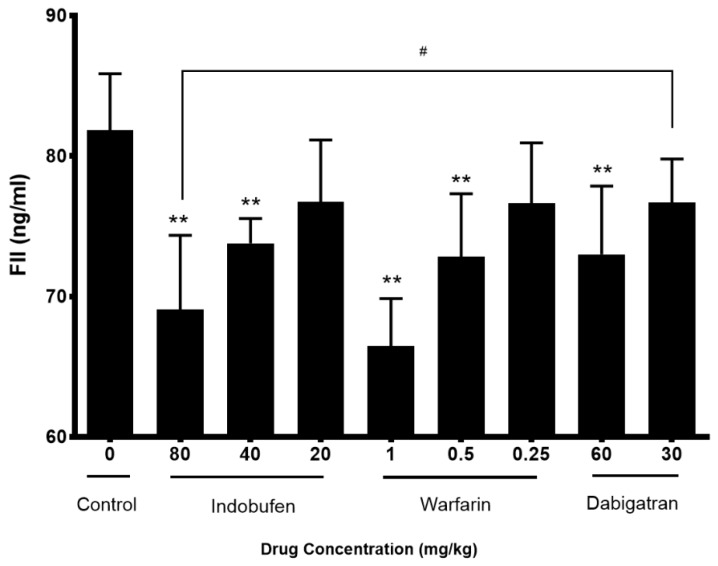
The effects of indobufen on the content of coagulation factor 2 (FII) in rats. ** *p* < 0.01 versus the control group; ^#^ *p* < 0.05, versus the dabigatran 30 mg/kg group (mean ± S.D., *n* = 10).

Figure 6 shows that compared with the negative control group, 80 and 40 mg/kg of indobufen, 1 and 0.5 mg/kg of warfarin sodium and 60 mg/kg dabigatran etexilate can all significantly reduce the content of clotting factor 2 (*p* < 0.01). The FIIa inhibitor dabigatran etexilate could significantly reduce the FII content and there was a significant difference between the 80 mg/kg indobufen and 30 mg/kg dabigatran groups (*p* < 0.05). 

### 2.5. Indobufen Reduced the Content of Coagulation Factor 10 in Rats

**Figure 7:**
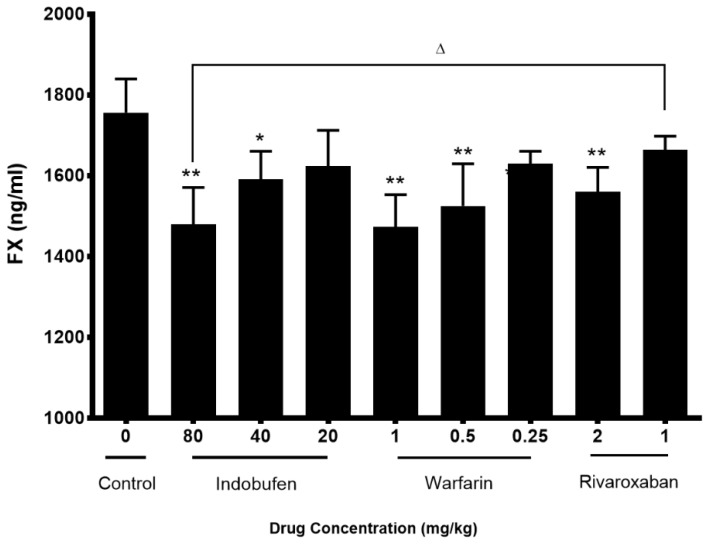
The effects of indobufen on the content of coagulation factor 10 (FX) in rats. * *p* < 0.05, ** *p* < 0.01 versus the control group; ^△^ *p* < 0.05 versus the rivaroxaban 1 mg/kg group (mean ± S.D., *n* = 10).

Figure 7 shows that compared with the negative control group, 80 and 40 mg/kg of indobufen, 1 and 0.5 mg/kg of warfarin sodium and 2 mg/kg of rivaroxaban can all significantly reduce the content of clotting factor 2 (*p* < 0.05). The FXa inhibitor rivaroxaban significantly reduced the FX content and there was a significant difference between the 80 mg/kg indobufen and 1 mg/kg rivaroxaban groups (*p* < 0.05).

## 3. Discussion

Although indobufen has been adequately studied on its anti-platelet aggregation efficacy, preclinical and clinical studies of its anticoagulant function and mechanisms are rare. Earlier studies showed that indobufen has antithrombotic effects, but no specific mechanism has been studied [24]. The existing clinical studies mainly focus on the antiplatelet effect of indobufen [25]. It was merely used as an adjunct to compare the antithrombotic effects of warfarin and aspirin in the research of antithrombotic effects [26]. In this study, we investigated the anticoagulation mechanism of indobufen.

The study of the coagulation system mainly includes the determination of platelet factors and coagulation factors in the intrinsic and extrinsic coagulation pathways [27]. The inhibition of platelet activation and coagulation is considered a strategy for the treatment of thrombotic diseases [28]. The detection of the anti-thrombotic effect is also a way to appraise the anticoagulant effects. Therefore, this research can be divided into three parts. 

We first investigated the effect of indobufen on the intrinsic and extrinsic coagulation pathways. PT often reflects the extrinsic coagulation system, mainly related to coagulation factors FII, V, VII, X and fibrinogen; and APTT mainly works as a screening test of the endogenous coagulation system. TT reflects the time when fibrinogen is converted to fibrin and is mainly used as a screening test for the fibrinolytic system [29]. Indobufen prolonged APTT, PT, and TT (*p* < 0.01), indicating that it promotes thrombin generation by acting on the pathway of both exogenous and endogenous coagulation systems while inhibiting the conversion of fibrinogen to fibrin network. This mechanism can be verified by the increased clotting and bleeding times in mice, in which 160 (*p* < 0.01) and 80 (*p* < 0.05) mg/kg of indobufen significantly reduced both the coagulation time and bleeding time and the effect was comparable to that of warfarin. The significantly prolonged bleeding time demonstrates that indobufen affects platelet and capillaries-related functions in mice; while significantly prolonged clotting time demonstrates that indobufen has anticoagulant effects and acts on endogenous anticoagulation. Blood system

In the second part, the anticoagulant study on rabbit plasma showed that indobufen can significantly reduce the in vitro plasma FI, II, V, VIII, X and PF3 contents, among which the effect of reducing PF3 was significantly better than warfarin sodium (*p* < 0.01). Preliminary analysis of the mechanism of anticoagulant activities showed that indobufen inhibits the catalytic activity of PF3 as a fixed site of FV, and decreases FV and FVIII to reduce the activation of FX and prothrombin (FII), eventually resulting in the decreased activation of fibrinogen (FI). In addition, the decrease of FII activation reduces the FV and FVIII feedback amplification by FIIa. Altogether, these processes result in the reduced function of endogenous coagulation systems.

The above studies initially confirmed that indobufen has some anticoagulant ability aside from its antiplatelet aggregation effects and the mechanisms are related with the platelet factor and coagulation factors in both exogenous and endogenous coagulation systems. However, compared with conventional anticoagulant warfarin sodium, indobufen has no obvious difference. Therefore, in order to explore the advantages of indobufen over clinically used anticoagulants, in the third part, we used the way of an arteriovenous bypass to form a thrombus model and picked three commonly used anticoagulant drugs as contrasts [1,30]. In this experimental model, indobufen can significantly reduce the dry weight and wet weight of thrombus, and 40 and 80 mg/kg of indobufen are both superior to 1 mg/kg of rivaroxaban (*p* < 0.01), proving its antithrombotic effect. For the key factors II and X in the coagulation systems, the 40 and 80 mg/kg indobufen groups are able to significantly reduce the content (*p* < 0.05), demonstrating their anticoagulant effect. Additionally, it is superior to the FIIa inhibitor dabigatran in reducing FII (*p* < 0.05), and superior to the FXa inhibitor rivaroxaban in reducing FX (*p* < 0.05). 

In summary, our studies show that indobufen gavage administration has a strong anticoagulant effect, and the mechanism may be related to platelet factor 3, and the release of the various coagulation factors in both exogenous and endogenous coagulation systems. Furthermore, the anticoagulant effect of indobufen is better than some conventional anticoagulants like rivaroxaban and dabigatran. Therefore, the pharmacological effects of indobufen in the coagulation system can have important clinical research value.

## 4. Materials and Methods 

### 4.1. Animals

Male and female New Zealand white rabbits (1.8–2.2 kg) were provided by the Qinglongshan Animal Breeding Farm (Nanjing, China); male and female Institute of Cancer Research (ICR) mice (18–22 g) were provided by the Yangzhou University Comparative Medical Center (Yangzhou, China); male and female Sprague Dawley (SD) rats (180–220 g) were provided by Shanghai Jieshi Experimental Animal Co., Ltd. (Shanghai, China). The experimental animal ethical approval number: SYXK(Su)2016-0011. All the animals were kept in a temperature-controlled environment (25 ± 2 °C) with 55 ± 5% relative humidity and a 12 h light-dark cycle, and fed with standard chow for at least 1 week before any manipulations. Before each experiment, all animals were fasted for 12 h in advance to rule out the effect of feed on thrombosis. All protocols were approved by the University Ethics Committee on Animal Research and conformed to National Institutes of Health Guideline for Care and Use of Laboratory Animals. Meanwhile, all experiments were carried out in accordance with the Guidelines for the Care and Use of Laboratory Animals and ethical approval was provided by the Animal Care and Use Committee of China Pharmaceutical University.

### 4.2. Reagents

Indobufen (C_18_H_17_NO_3_), warfarin sodium, rivaroxaban, and dabigatran etexilate were all provided by Hangzhou Zhongmei Huadong Pharmaceutical Co., Ltd. (Hangzhou; China). For gavage administration, the above medicines were dissolved in 500 μL of DMSO and diluted with 0.9% saline injection. All other reagents were of analytical grade and commercially available. Since the recommended dose for indobufen is 100–200 mg bid [25], in rabbits, the dose is designed to be 20 mg/kg, and the mice dosages are designed to be 160, 80, and 40 mg/kg. The dosages are 80, 40, and 20 mg/kg in rats. The kits of APTT, PT, and TT were provided by the Beijing World Science Instrument Co., Ltd. (Beijing, China); the kits of platelet factor 3, 4, and the kits of clotting factors 1, 2, 5, 8, 10 were provided by the Nanjing Institute of Bioengineering Research Institution (Nanjing, China).

### 4.3. Plasma Coagulation Factors Measurement in Rabbits 

Rabbits (*n* = 10) were randomly divided into 3 groups, 3 for the positive control warfarin sodium group (0.25 mg/kg), 3 for the indobufen group (20 mg/kg), and 4 for the negative control group (normal saline). The administration volume was 1 mL/kg, once a day for 5 days. Thirty minutes after the last administration, the carotid artery cannulation blood was collected into a test tube containing 3.8% sodium citrate and centrifuged at 1000 rpm for 10 min. The supernatant was discarded to get platelet-rich plasma (PRP). Then the pellet was centrifuged at 3000 rpm for 5 min and the supernatant is the poor platelet plasma (PPP) [31]. PT, APTT, and TT were determined according to the kit instructions. Another part was centrifuged at 2500 rpm for 10 min and the upper plasma was used to test the platelet factor 3, 4 and clotting factor 1, 2, 5, 8, 10 according to the kit instructions.

### 4.4. Bleeding and Clotting Time Measurement in Mice

ICR mice (*n* = 50) were divided randomly into 5 groups of 10 mice each: the negative control group (saline), positive control warfarin (1 mg/kg), and indobufen (160 or 80 or 40 mg/kg) groups. Each group was administered once a day for 5 consecutive days. The bleeding time was evaluated via the method devised by Seiji Kaku et al. (2013) with some modifications [32,33,34] Thirty minutes after the last administration, the caudal parts were cut perpendicularly at a distance of 5 mm from the caudal end. We started counting every 30 s when the blood spilled out. The bleeding time was recorded with a stopwatch until the blood flow stopped naturally (no blood was drawn on the filter paper). The clotting time was tested in the similar way with an inner diameter of 1 mm glass capillary inserted into the orbit of the mouse. The first drop of blood was discarded, and then they were splashed on both ends of the clean glass slide, with the blood droplet diameter of 5~10 mm. Then we stirred the blood every 10 s with a dry needle until the needle picked up the fibrous protein and recorded that time as the clotting time.

### 4.5. Arteriovenous Shunt Thrombosis Model in Rats

To investigate the effect of indobufen on the fibrinolysis system and to compare the anti-thrombotic effects of indobufen with commonly used anticoagulants, we used the arteriovenous bypass model and compared indobufen with warfarin sodium, rivaroxaban, and dabigatran on the thrombus inhibition rate. SD rats (*n* = 110) were randomly divided into 11 groups and given rivaroxaban (2 or 1 mg/kg, i.g.) [20], dabigatran etexilate (60 or 30 mg/kg, i.g.) [21], warfarin sodium (1 or 0.5 or 0.25 mg/kg, i.g.) [35], and indobufen (80 or 40 or 20 mg/kg, i.g.) or an equal volume of saline separately. The administration volume was 1 mL/kg, once a day for 5 days. Thirty minutes after the last administration, the rats were anesthetized with 3% chloral hydrate (1 mL/kg, i.p.), and the right common carotid artery and the left external jugular vein were separated. Taking a polyethylene tube with an inner diameter of 1.5 mm and a length of 10 cm, a 13 cm long pre-weighed filament inserted in the polyethylene tube was pre-filled with a heparin physiological saline solution (50 U/mL). We inserted one end of the polyethylene tube into the left external jugular vein and the other into the right common carotid artery. We interrupted the blood flow after 15 min and quickly removed the thread. The total weight of the thread minus the original thread weight is the wet thrombus weight. The total weight minus the thread weight is the dry thrombus weight after the thread was dried at 70 °C to a constant weight. We calculated the inhibition rate of both wet and dry thrombus weight [34]

$$\mathrm{Inhibition}\;\mathrm{rate}=\frac{\mathrm{Control}\;\mathrm{group}\;\mathrm{weight}-\mathrm{Administration}\;\mathrm{group}\;\mathrm{weight}}{\mathrm{Control}\;\mathrm{group}\;\mathrm{weight}}\times 100\%$$

### 4.6. Coagulation Factors 2 Measurement in Rats

To investigate the effect of indobufen on the coagulation system and to compare the anticoagulant effects of indobufen with commonly used anticoagulants, we further compared indobufen with warfarin sodium and FIIa inhibitor dabigatran etexilate on plasma FII content. The SD rats (*n* = 90) were randomly divided into 9 groups and administered dabigatran etexilate (60 or 30 mg/kg, i.g.), warfarin sodium (1 or 0.5 or 0.25 mg/kg, i.g.), and indobufen (80 or 40 or 20 mg/kg, i.g.) or an equal volume of saline separately. The administration volume was 1 mL/kg, once a day for 5 days. Then the PPP was collected as mentioned in Section 2.3 and assayed according to the instructions of the kit for FII content.

### 4.7. Coagulation Factors 10 Measurement in Rats

We compared indobufen with warfarin sodium and FXa inhibitor rivaroxaban on the plasma FX content. SD rats (*n* = 90) were randomly divided into 9 groups and administered rivaroxaban (2 or 1 mg/kg, i.g.), warfarin sodium (1 or 0.5 or 0.25 mg/kg, i.g.), and indobufen (80 or 40 or 20 mg/kg, i.g.) or an equal volume of saline separately. The administration volume was 1 mL/kg, once a day for 5 days. Then PPP was collected as mentioned in Section 4.3 and assayed according to the instructions of the kit for FX content.

### 4.8. Statistical Analysis

All data are expressed with mean ± the standard errors of the mean (SEM). One way ANOVA was done followed by Tukey’s post hoc test. Unpaired *t*-test was used for comparisons between groups. A minimum level of significance is considered if *p* is <0.05.
